# Suramin Interactions Across Biological Systems: From Molecular Targets to Therapeutic Implications

**DOI:** 10.3390/biom16040527

**Published:** 2026-04-01

**Authors:** Alessia Catalano, Valeria Scaglione, Maria Noemi Sgobba, Lavinia Ferrone, Anna Lucia Francavilla, Maria Maddalena Cavalluzzi, Sabino Todisco, Lorenzo Guerra, Mariateresa Volpicella, Anna De Grassi, Giovanni Lentini, Ciro Leonardo Pierri

**Affiliations:** 1 Department of Pharmacy—Pharmaceutical Sciences, University of Bari “Aldo Moro”, Via E. Orabona 4, 70125 Bari, Italy; alessia.catalano@uniba.it (A.C.); mariamaddalena.cavalluzzi@uniba.it (M.M.C.); giovanni.lentini@uniba.it (G.L.); 2 Department of Biosciences, Biotechnologies and Environment, University of Bari “Aldo Moro”, Via E. Orabona 4, 70125 Bari, Italy; v.scaglione@phd.uniba.it (V.S.); maria.sgobba@uniba.it (M.N.S.); anna.francavilla@uniba.it (A.L.F.); s.todisco11@phd.uniba.it (S.T.); lorenzo.guerra1@uniba.it (L.G.); mariateresa.volpicella@uniba.it (M.V.); anna.degrassi@uniba.it (A.D.G.); 3 Divisione di Oncologia Medica, A.O.U. Consorziale Policlinico di Bari, Piazza Giulio Cesare 11, 70124 Bari, Italy; lavinia.fer@gmail.com; 4 Dipartimento Interdisciplinare di Medicina, Università degli Studi di Bari “Aldo Moro”, Piazza Giulio Cesare 11, 70124 Bari, Italy; 5 Laboratory of Biochemistry, Structural, and Molecular Biology, Department of Pharmacy—Pharmaceutical Sciences, University of Bari “Aldo Moro”, Via E. Orabona 4, 70125 Bari, Italy

**Keywords:** suramin, polypharmacology, mitochondrial carriers, ADP/ATP carrier (AAC), SLC25 family, aspartate/glutamate carrier, mitochondrial metabolism, structural biology, protein–ligand interactions, purinergic receptors, antiparasitic agents, drug repurposing

## Abstract

Suramin is a century-old polysulfonated naphthylurea that remains a first-line treatment for early-stage human African trypanosomiasis (HAT). Remarkably, despite its age, suramin continues to draw attention because of its unusually broad spectrum of biological activities. Historically known as an antagonist of purinergic (P2) receptors and an inhibitor of extracellular enzymes, suramin has more recently been shown to interact with a range of intracellular and mitochondrial proteins. These include succinate dehydrogenase, the ADP/ATP carrier (AAC), the aspartate/glutamate carriers AGC1 and AGC2, carnitine O-acetyltransferase (CRAT), and the ATP-Mg/Pi carrier (APC2). Across these targets, suramin displays sub-micromolar to low-micromolar potencies, largely driven by electrostatic complementarity between its highly anionic sulfonate groups and basic nucleotide- or anion-binding regions of proteins. This extensive polypharmacology helps explain the diverse biological effects reported for suramin and supports its use as a valuable pharmacological probe of mitochondrial transport and metabolism. At the same time, its largeness and high negative charge limit oral bioavailability and brain penetration, prompting efforts to develop simplified analogues. This review brings together chemical, biological, and structural perspectives on suramin, highlighting opportunities for drug repurposing, transporter-focused drug design, and a better understanding of mitochondrial toxicity.

## 1. Introduction

**Figure 1:**
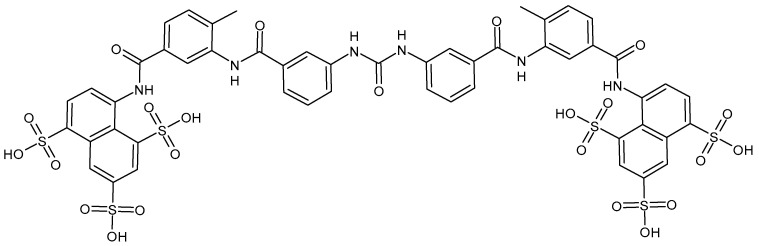
Structure of suramin.

Discovered in 1916 by Bayer as the first effective synthetic treatment for HAT, suramin (Germanin^®^, Figure 1) has endured as one of the oldest continuously used drugs in medicine [1,2]. Chemically, it is a symmetric hexasulfonated [naphthylaminocarbonyl(tolylaminocarbonyl)]phenylurea that combines high aqueous solubility with a molecular weight exceeding 1.4 kDa. It also belongs to the class of diarylureas, a privileged scaffold in medicinal chemistry [3], specifically a symmetric diarylurea containing multiple anionic groups. Its polyanionic nature precludes passive diffusion across biological membranes, but allows extremely tight binding to serum proteins and positively charged protein surfaces. Early clinical success against parasitic infections such as *Trypanosoma brucei* and *Onchocerca volvulus* established suramin as a mainstay of therapy for neglected tropical diseases.

For decades, suramin’s mechanism of action was thought to rely primarily on inhibition of extracellular purinergic receptors and enzymes critical for nucleotide supply in parasites. Suramin has been studied as a treatment for several diseases, and has been shown to bind to diverse proteins, including the spike protein and RNA-dependent RNA polymerase (RdRp) of SARS-CoV-2 [4,5,6,7], Zika virus (ZIKV) [8], alpha-thrombin [9], *Leishmania mexicana* pyruvate kinase [10,11], human sirtuin homolog 5 (SIRT5) [12], and Ebola virus polymerase complex [13]. However, its unusually broad range of biological effects, spanning antiviral, antitumor, and neuromodulatory activities, hinted at a much wider molecular footprint, and suramin is not listed in any PAINS database [14]. Remarkably, renewed investigation in the post-genomic era, aided by high-throughput screening and improved mitochondrial protein expression systems, has unveiled a complex network of intracellular targets. More recently, suramin has emerged as an unexpected modulator of mitochondrial function. Biochemical and structural studies have shown that beyond its well-established extracellular and cytosolic targets, suramin can interact with multiple mitochondrial proteins involved in energy metabolism (i.e., CRAT, SIRT5 and SDH, Refs. [12,15,16]) and metabolite transport [2,17,18,19,20,21]. These findings have renewed interest in suramin as a pharmacological probe for mitochondrial pathways and have raised important questions regarding both its therapeutic actions and side-effect profile. Its electrostatic multivalency allows high-affinity binding to nucleotide-binding proteins, but also underlies its notorious lack of selectivity and poor pharmacokinetics. Only a limited number of clinical studies are reported for suramin, specifically regarding the treatment of autism spectrum disorder (ASD) and some types of cancers [22,23]. Several analogues of suramin have been studied in the literature. Understanding how suramin recognizes and inhibits its various targets through structural biology and computational docking approaches offers a rational basis for developing smaller and more selective analogues. The present review synthesizes current knowledge on suramin’s chemical structure, its interactions with mitochondrial carriers and other enzymatic targets, and the implications of these findings for drug discovery and therapeutic repurposing. In addition, this review attempts to bridge classical medicinal chemistry and contemporary structural biology to explain how a century-old drug continues to reveal new insights into molecular pharmacology. The present review aims to integrate recent advances in structural biology, mitochondrial biochemistry, and cross-species sequence analysis in order to reinterpret suramin polypharmacology within a unified mechanistic framework. In particular, the structural determinants of suramin binding across diverse protein families are emphasized, and how these interactions may involve both mitochondrial pharmacology and cross-species target prediction is discussed. Taken together, these perspectives position suramin not only as a historically important antiparasitic drug but also as a valuable molecular probe to investigate conserved structural determinants of ligand recognition across mitochondrial transporters and other protein families. A unifying feature emerging from these observations is that suramin’s polypharmacology appears to be largely driven by its electrostatic multivalency: the highly polyanionic scaffold of the molecule enables interaction with extended electropositive surfaces and nucleotide- or anion-binding regions present in many structurally unrelated proteins.

## 2. Chemical Description, Physicochemical Properties, and Synthetic Routes to Suramin

### 2.1. Chemical Structure and Salient Features

Suramin (8,8′-carbonylbis[imino-3,1-phenylencarbonylimino(4-methyl-3,1-phenylen)carbonylimino]]bis-1,3,5-naphthalentrisulfonic acid, Figure 1; C_51_H_40_N_6_O_23_S_6_; 1.4 kDa) is a large, highly anionic molecule (multiple sulfonate groups), formally highly polar and hydrophilic, typically used as the hexasodium salt for aqueous administration. This polyanionic character underlies both its multimodal protein binding and poor membrane permeability. Structurally, suramin is a polysulfonated [naphthylaminocarbonyl(tolylaminocarbonyl)]phenylurea, with a comparable high molecular weight (1297.29 g/mol) [24] for a small molecule and carrying negative charges based on its sulfate groups, allowing it to interact with a broad range of biological targets [1]. Its very high plasma protein binding, very long terminal half-life (weeks), and low CNS penetration mean that intravenous injection is the only administration route. These properties influence achievable free concentrations in tissues and therefore the feasibility of engaging intracellular/mitochondrial targets. The structure of suramin resembles the widely known glycosaminoglycan (GAG) heparin, with structural and physiological similarities as negatively charged polymers and high sulfation levels. Thus, suramin is often considered an analogue of heparin or heparinoid [25]. However, despite its therapeutic potential, suramin is limited by its poor cell permeability, the need for intravenous administration, and its toxicity [1]. Its binding to diverse proteins further contributes to off-target effects, which limit its broader clinical use.

### 2.2. Synthetic Routes to Suramin

**Scheme 1:**
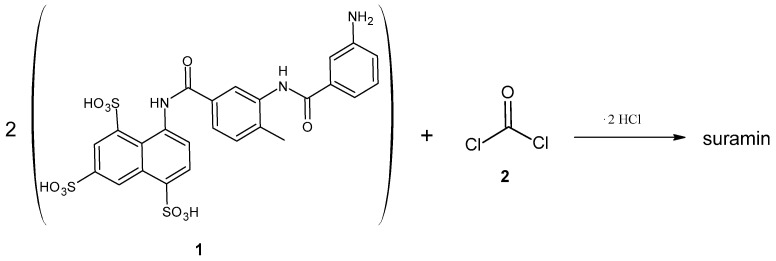
Synthesis of suramin by Dressel (1961) [26].

The first synthesis of suramin was reported by Dressel in 1941 [26], and recently reviewed by Steverding and Troeberg (2024) [1] (Scheme 1). Suramin is obtained by reaction of *m*-aminobenzoyl-3-amino-4-toluoyl-1-naphthylamine-4,6,8-trisulfonic acid (**1**) with phosgene (**2**). This compound was initially named Bayer 205.

**Scheme 2:**
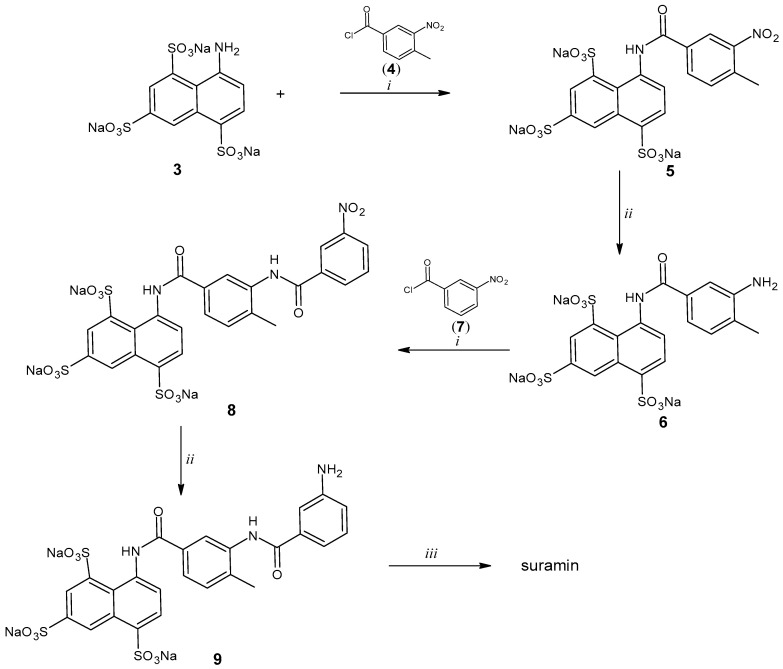
Synthesis of suramin by Ullman et al. (2005) [29] and Calder et al. (2022) [30]. Reaction conditions: (i) pH = 4 or Na_2_CO_3_, toluene/H_2_O; (ii) H_2_, Pd/C (or Raney nickel), H_2_O; (iii) phosgene, toluene/H_2_O or 1,1-carbonyldiimidazole, dichloromethane/dimethylformamide.

The first complete synthesis of suramin, as the sodium salt, was described by Nickel et al. (1986) [27] in an article in German, and detailed by the same group in 2004 [28] and 2005 [29] (Scheme 2). Sodium 8-aminonaphthalene-l,3,5-trisulfonate (**3**) was reacted with 4-methyl-3-nitrobenzoyl chloride (**4**). The nitro group of the nitrobenzoic derivative obtained (**5**) was reduced by activated iron to an amino group (**6**), which was then acylated by *m*-nitrobenzoylchloride (**7**) to give a new nitroderivative, 8-(4-methyl-3-(3-nitrobenzamido)benzamido)naphthalene-1,3,5-trisulfonic acid (**8**), which is in turn reduced to amine (**1**). The reaction of the resulting product (**1**) with phosgene gives sodium [8,8′-carbonylbis[imino-3,1-phenylencarbonylimino-(4-methyl-3,1-phenylen]carbonylimino]bis-1,3,5-naphthalenetrisulfonate. Ullmann’s group [29] did not report the yield of the last coupling step (generally allowing for moderate to good yields). Thus, an overall yield ≤ 53% might be estimated for their method. A recent patent by Calder et al. (2022) [30] describes an alternative synthesis of suramin and is also depicted in Scheme 2.

Specifically, in a synthesis by Calder et al. [30], sodium 8-aminonaphthalene-l,3,5-trisulfonate (**3**) was reacted with 4-methyl-3-nitrobenzoyl chloride (**4**) in a mixture of toluene and water to give sodium 8-(4-methyl-3-nitrobenzamido)naphthalene-l,3,5-trisulfonate (**5**), as an aqueous solution, which was carried forward to the next synthetic step without further purification. The crude solution was treated with Pd/C (or Raney nickel), and the reactor sealed up and connected to pressurized nitrogen and hydrogen sources. Upon complete consumption of the starting material, the reaction mixture was filtered to obtain sodium 8-(3-amino-4-methylbenzamido)naphthalene-l,3,5-trisulfonate (**6**). The resulting aqueous solution was carried forward to the next synthetic step without further purification. The crude solution was reacted with 3-nitrobenzoyl chloride (**7**) in a mixture of toluene and water (the pH of the aqueous layer was monitored and maintained above pH 2.0 via addition of sodium carbonate solution) to give sodium 8-(4-methyl-3-(3-nitrobenzamido)benzamido)naphthalene-1,3,5-trisulfonate (**8**). The resulting aqueous solution was carried forward to the next synthetic step without further purification. Reduction with Pd/C or Raney nickel in water afforded sodium 8-(3-(3-aminobenzamido)-4-methylbenzamido)naphthalene-l,3,5-trisulfonate (**9**). The salt was reacted with imidazole hydrochloride and suspended in 4:1 acetonitrile/water. Then, 1,1-carbonyldiimidazole, which is a safer and easier to handle acyl transfer agent than phosgene [31], was added to afford crude suramin, which was purified to afford suramin in an overall yield of approximately 54%.

The two main synthetic routes described in the literature appear comparable, considering their respective yields. From an atom economy point of view, Ullmann’s procedure seems to be slightly preferable, but Calder’s route is more practical.

## 3. Biological Contexts and Molecular Targets of Suramin

Its chemical and pharmacokinetic properties underpin suramin’s ability to interact with a wide spectrum of biological targets, as discussed in the following sections.

### 3.1. Extracellular and Plasma Membrane Targets in Biological Contexts

**Figure 2:**
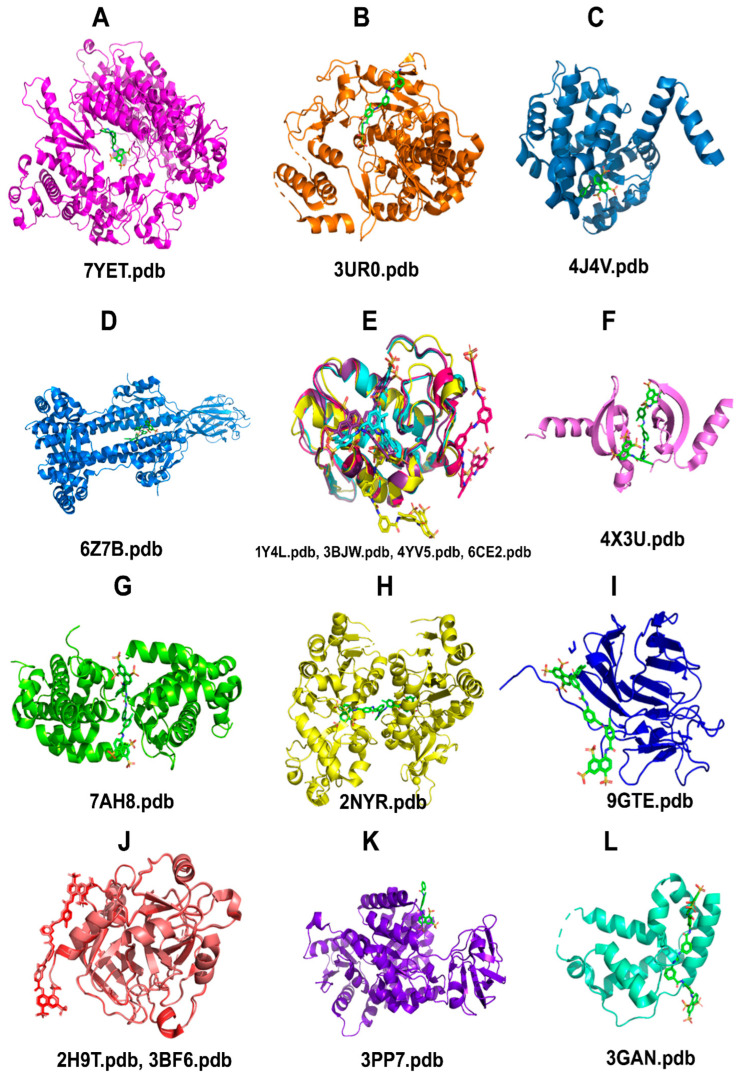
Representative crystal structures of proteins crystallized in complex with suramin. The figure illustrates the diversity of suramin-binding partners across viral, parasitic, enzymatic, and regulatory systems. (**A**,**B**) RNA-dependent RNA polymerases from RNA viruses (PDB: 7yet, 3ur0). (**C**) Viral nucleocapsid protein (PDB: 4j4v). (**D**) Variant surface glycoprotein VSGsur from *Trypanosoma*, associated with altered suramin sensitivity (PDB: 6z7b). (**E**) Snake venom toxins structurally related to the phospholipase A_2_ (PLA_2_) family (PDB: 1y4l, 3bjw, 4yv5, 6ce2). (**F**) Chromobox (CBX) chromodomains involved in polycomb-mediated chromatin regulation (PDB: 4x3u). (**G**) Nuclear transcription factor Y (NF-Y) subunits (PDB: 7ah8). (**H**) Sirtuin 5 (SIRT5), an NAD^+^-dependent deacylase (PDB: 2nyr). (**I**) PRY/SPRY domain of the E3 ubiquitin ligase TRIM21 (PDB: 9gte). (**J**) Thrombin, a serine protease involved in coagulation and inflammatory signaling (PDB: 2h9t, 3bf6). (**K**) Pyruvate kinase from *Leishmania* species, a glycolytic enzyme involved in parasite metabolism (PDB: 3pp7). (**L**) Plant protein illustrating conserved suramin-binding motifs (PDB: 3gan). Across these complexes, suramin preferentially binds extended positively charged surfaces and nucleotide- or anion-binding regions. When combined with sequence conservation analysis (Table S1), these structures provide a framework for predicting potential suramin interactions across homologous proteins and biological contexts.

Given the polyanionic nature of suramin, it can bind with a diverse array of biomacromolecules and display different activities, such as antiviral, anticancer, anti-inflammatory, antifibrotic, nephroprotective [32], and antiparasitic properties [1,2]. It was also used for onchocerciasis [33,34], and it has been proposed as a protective agent against liver or kidney damage [35], therapy for arthritis and autism [22], and an antidote for snake bites [36,37]. In addition, suramin has been recently suggested as an important drug in diabetic kidney disease (DKD) [35,38]. It has also been recently demonstrated that suramin stimulates adipocyte differentiation and promotes adipogenesis [39]. Recent investigations identify suramin as a novel HIV inhibitor that synergizes with MLN4924 to enhance therapeutic efficacy [40]. The antiviral activity against lymphocytic choriomeningitis virus (LCMV), a prototype of the pathogenic human arenaviruses, is under study [41]. Eberle et al. (2021) [42] suggested that suramin, in association with quinacrine, showed promising synergistic efficacy to inhibit SARS-CoV-2 3CLpro for the treatment of COVID-19. Suramin antiviral activity has recently been supported by structural studies capturing suramin bound to viral structural proteins (Figure 2). The role of suramin in association with sodium phenylbutyrate has been studied in the prevention of ulcerative colitis induced by dextran sulfate sodium [43]. Moreover, suramin has been suggested as a promising adjunct to 1,25-dihydroxyvitamin D3-based immunotherapy for hepatocellular carcinoma [44]. Suramin has recently been suggested for the treatment of rotator cuff tendinopathy, a common musculoskeletal disorder with limited pharmacological treatment strategies [45]. Suramin has also been suggested in mitigating AD neuronal pathology, due to its autophagy modulation properties [46]. Finally, the therapeutic potential of suramin in alleviating fibromyalgia symptoms has been reported by Mohamed et al. [47] and in intestinal inflammation by Ercan et al. (2025) [48].

Ebbert et al. (2025) [49] suggested the usefulness of suramin in TNF⍺-mediated mast cell and brain microvascular endothelial cell (BMEC) dysfunction, a condition that may lead to chronic blood–brain barrier (BBB) disruption, commonly observed in neuroinflammatory and neurodegenerative conditions. A recent study suggests the potential of suramin for the treatment of papillary thyroid carcinoma [50]. Moreover, the possible use of suramin as a medicine for the treatment of neointimal hyperplasia has been described [51]. Suramin has been studied as an alternative inducer for specific cardiomyocyte lineage commitment during human cardiomyocyte induction processes. By activating ryanodine receptor 2 (RyR2), it efficiently promoted cardiac differentiation even in the absence of conventional WNT inhibitors [52]. Finally, suramin has been suggested as a new treatment approach for mitigating the effects of chronic stress on restoring neurobehavioral changes [53]. Collectively, these observations indicate that suramin predominantly interferes with extracellular signaling systems by targeting proteins involved in nucleotide recognition and growth-factor signaling, illustrating how its polyanionic structure enables interactions with diverse regulatory pathways. Remarkably, while the presented studies illustrate the wide range of biological processes influenced by suramin, many of the reported disease contexts derive from exploratory or preclinical investigations, and therefore their translational relevance should be interpreted cautiously in the light of the compound’s pharmacokinetic limitations and toxicity profile.

### 3.2. Intracellular Targets

#### 3.2.1. Enzymes and Signaling Proteins

Several targets have been proposed for the biological activities of suramin (Table 1). It inhibits various types of ATPases (mitochondrial ATPase, vacuolar H^+^-ATPases), GTPase, DNA and RNA polymerases, and reverse transcriptase [2], and can also inhibit the binding of platelet-derived growth factor (PDGF), fibroblast growth factor (FGF), epidermal growth factor (EGF), insulin-like growth factor (IGF-I), and tumor growth factor beta (TGF beta) to their receptors [54]. The function of several cellular signaling proteins, such as protein-tyrosine phosphatases [55] and protein kinase C [56], is perturbed, and it interferes with signaling through G protein-coupled receptors (GPCRs) [57]. It should be noted that the strength of evidence supporting these interactions varies considerably across the literature, ranging from crystallographic structures and direct biochemical inhibition assays to cellular observations and computational predictions. To help the reader navigate this heterogeneous evidence, targets for which direct structural evidence of suramin binding is available are discussed separately in Section 3.4.

The most studied activity of suramin is the antagonistic action against purinergic receptors. The inhibition of P_2_X purinoceptors in the guinea-pig urinary bladder and P_2_Y purinoceptors in the guinea-pig taenia coli, respectively, was reported at the end of last century [58,59]. Suramin antagonizes most homomeric P2X subtypes at low micromolar concentrations, except for the P2X7 receptor, where hundreds of micromolar suramin are required, and the P2X4 receptor, which is insensitive [60]. It is non-selective against P2Y receptors, and inhibits the P2Y_2_R with moderate potency (IC_50_ according to literature ca. 50 μM) [61]. Chanalaris et al. (2017) [62] found that suramin binds to tissue inhibitor of metalloproteinases 3 (TIMP3) with a *K*_d_ value of 1.9 ± 0.2 nM and inhibited its endocytosis via LRP1, thus increasing extracellular levels of TIMP3 and inhibiting cartilage degradation by the TIMP3 target enzyme, adamalysin-like metalloproteinase with thrombospondin motifs (ADAMTS)-5, a group of metalloproteinases involved in cartilage matrix degradation by degrading aggrecan, thus studied for the treatment of osteoarthritis. Recent studies have focused on different targets. It has been recently suggested that suramin acts as an inhibitor of NLRP-3/CASPASE-1-mediated inflammasome activation in macrophages [63], and has been shown to bind and inhibit the cullin–RING E3 ubiquitin ligase by disrupting its ability to recruit the E2 enzyme Cdc34 [64].

The potential usefulness of suramin in thyroid cancer was studied by Van Branteghem et al. (2025) [50], who demonstrated that HMGA2 inhibition by suramin reduced cell invasion and induced differentiation expression in vitro. In colorectal cancer, Zaragoza-Huesca et al. (2023) [65] showed that suramin reduces the prothrombotic and metastatic phenotypes of colorectal cancer cells, acting as a potent hepsin inhibitor, reducing the protumoral effects of hepsin in vitro, decreasing hepsin-mediated thrombin generation, and suppressing the invasive effects of hepsin in a zebrafish model. A recent molecular modelling study identified suramin as a tripartite motif-containing protein 21 (TRIM21) binder [66]. Using differential scanning fluorimetry screening of the Library of Pharmacologically Active Compounds (LOPAC), four hits for the PRYSPRY domain of the human E3 ligase TRIM21 were identified. Suramin was found as a binder with micromolar affinity, as confirmed by isothermal titration calorimetry. To further investigate the binding mechanism, mouse TRIM21 was used as a structural surrogate due to its improved protein stability and high sequence similarity to the human counterpart. A crystal structure of the complex refined at 1.3 Å resolution revealed a unique binding mode, providing new avenues for targeting TRIM21 and for the development of proteolysis-targeting chimeras (PROTACs). The crystal structure of the mouse TRIM21–suramin complex suggested the possibility of substituting them with less charged groups, such as sulfonamides or sulfonyl groups, which would likely increase cell permeability. Furthermore, the authors suggested that suramin, having a symmetrical structure, may be reduced to a ligand of half its size with minimal modifications, providing a foundation for further exploration and validation. Moreover, they suggested an alternative promising strategy that may focus on covalently targeting the cysteine inside the small cavity formed by the outward movement of the C-terminus, involving a smaller version of the ligand equipped with a covalent warhead, such as an acrylamide, as commonly used in the development of covalent inhibitors [67]. Remarkably, suramin has been shown to antagonize other peptide receptor systems. Notably, Jenkinson and Reid demonstrated that suramin acts as a competitive antagonist at vasoactive intestinal peptide (VIP) receptors in the rat gastric fundus, consistent with its ability to interfere with peptide-binding sites through electrostatic interactions [68]. Beyond direct antagonism of purinergic and peptide receptors, suramin’s modulation of extracellular signaling is likely to reverberate across interconnected plasma membrane receptor networks. Many of the pathways influenced by suramin, including purinergic receptors, cytokine signaling (e.g., TNFα [69,70]), and T-cell receptor–associated immune responses [71,72,73,74,75], are functionally integrated with guidance and patterning receptors such as plexins. In particular, Plexin-D1, a semaphorin receptor implicated in vascular development, immune regulation, and tumor progression, participates in signaling cross talk with growth factor and cytokine pathways, illustrating how perturbation of selected receptor systems by suramin may indirectly influence broader cell-surface signaling architectures [76,77]. This network-level perspective aligns with current models of semaphorin/plexin signaling, in which receptor interactions extend beyond single ligand–receptor pairs to shape coordinated cellular responses. Taken together, the presented studies highlight how suramin modulates diverse signaling pathways through interactions with proteins involved in nucleotide recognition and regulatory signaling networks. These observations reinforce the concept that suramin recognition often relies on electrostatic complementarity between its multiple sulfonate groups and positively charged protein surfaces, particularly within nucleotide-binding cavities or regulatory interaction interfaces.

#### 3.2.2. Cytokines, Chemokines, and Inflammatory Pathways

In a study by Huang et al. (2025) [45], it was demonstrated that suramin ameliorated the proliferation, cell viability, and migration of tenocytes obtained from human supraspinatus tendons and enhanced the protein expression of PCNA and Ki-67. Suramin-treated tenocytes exhibited increased expression of collagen genes COL1A1 and COL3A1, tenascin C (TNC), scleraxis (SCX), and vascular endothelial growth factor (VEGF) genes. In addition, suramin significantly reduced LPS-induced iNOS, COX2 synthesis, inflammatory cytokine TNFα production, and inflammatory signaling [69,70,73,78] by influencing the NF-κB pathways in THP-1 cells. Recently, a potential interaction of suramin with thalamic P2X receptors and NLRP3 inflammasome activation has alleviated reserpine-induced fibromyalgia-like symptoms in the rotarod and open-field tests [47]. Suramin ameliorated the heightened pain processing, as observed in mechanical and thermal pain tests. The involvement of the NLRP3 inflammasome has also been studied in a diabetic kidney (DKD), showing that inhibition of the NLRP3 inflammasome with suramin protects against the progression of early-stage DKD. Moreover, suramin has been recently suggested as a potential interactor of IL8 on the basis of molecular modeling and biophysical studies. It was found that suramin interacts with IL8 with a dissociation constant of 3.02 ± 0.4 μM. Moreover, the overlapping binding site of suramin on IL8 in the receptor/glycosaminoglycan (GAG)-binding pocket was established by structural data. In addition, stability, dynamics, and conformity of IL8–suramin interactions were found to be influenced by structural plasticity and heterogeneity. Suramin has been proposed as an inhibitor of chemokine–GAG and chemokine–receptor interactions, thereby potentially modulating IL8-driven inflammatory responses [79]. It downregulates the expression of two major chemokines, CCL2 and CCL5, thus reducing glomerular/vascular injury-associated inflammation [80]. In addition, suramin inhibits the inflammatory enzyme heparanase (HPSE) [81], which hydrolyses heparan sulfate, an activity suggested as one possible contributor to its reported effects in COVID-19–related settings. In this regard, Tripathi et al. [25] characterized the CCL2–suramin complex and showed that suramin inhibits monocyte–macrophage migration. Suramin binds CCL2 with nanomolar affinity, and the interacting residues map to both the GAG-binding and receptor-binding regions on the chemokine surface, supporting a mechanism in which suramin disrupts CCL2 interactions required for chemotactic signaling. These findings further reinforce its potential for repurposing in inflammation-associated indications. Ebbert et al. [49] evaluated suramin (100 µM) as an Akt/mTOR/GSK pathway modulator, comparing its effects with rapamycin (250 nM) in TNFα-mediated mast cell and brain microvascular endothelial cell (BMEC) dysfunction. Suramin significantly decreased extracellular prostaglandin E2 (PGE2), granulocyte–macrophage colony-stimulating factor (GM-CSF), CCL2, and CCL5 while markedly increasing the blood–brain barrier (BBB), stabilizing platelet-derived growth factor BB (PDGF-BB). Notably, suramin also increased intracellular BMEC levels of multiple proinflammatory cytokines, indicating a mixed inflammatory profile that may depend on compartment and readout. Mechanistically, Zhang et al. (2020) [40] suggested that suramin interferes with the biosynthesis of inositol hexakisphosphate (IP6), a critical cofactor for viral capsid assembly. Suramin suppresses IP6 production by targeting IP5 kinase (IP5K), inhibiting ATP binding and IP5 phosphorylation. Reduced IP6 levels impair COP9 signalosome recruitment to cullin–RING ligases (CRLs), thereby disrupting the dynamic equilibrium required for CRL activity cycling. More recently, Zhang et al. (2025) [44] reported that suramin blocks hCAP18/LL-37–induced macrophage recruitment and M2 polarization, enhancing the therapeutic efficacy of 1,25(OH)2D3 against hepatocellular carcinoma in vitro and in vivo in a mouse model. In this framework, suramin disrupts the 1,25(OH)2D3–LL-37–tumor-associated macrophage (TAM) axis by limiting LL-37–mediated TAM recruitment and M2 polarization while promoting antitumor M1-type responses. Consistent with broader anti-inflammatory activity, Sharma et al. (2025) [53] showed that suramin protects against chronic stress–induced neurobehavioral deficits via suppression of cGAS–STING/NF-κB signaling: it dose-dependently improved neurobehavioral parameters, decreased corticosterone, elevated neurotransmitter levels, and mitigated oxidative stress and neuroinflammation in a chronic unpredictable stress model. Because of its polyanionic character, suramin can behave as a nucleotide analogue through electrostatic recognition of positively charged substrate cavities. This property provides a unifying rationale for its broad—yet still target-dependent—binding to nucleotide-binding and nucleotide-transport sites across diverse protein classes.

### 3.3. Mitochondrial Targets

#### 3.3.1. Mitochondrial Carriers of the Inner Membrane

**Figure 3:**
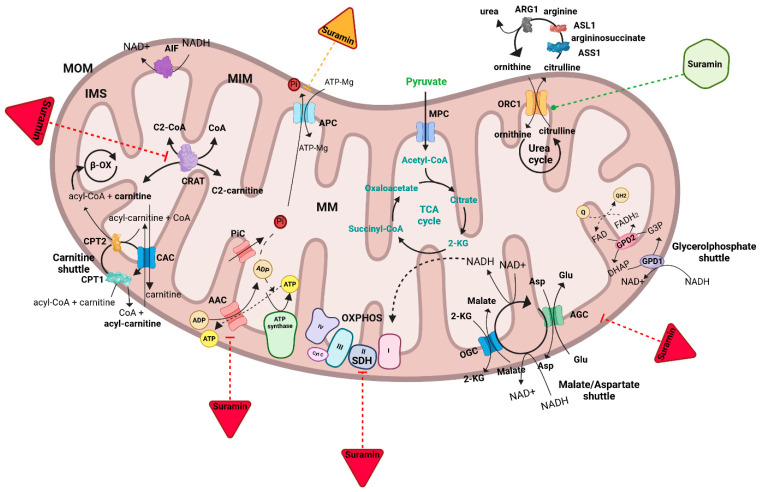
Schematic overview of mitochondrial metabolic pathways and inner mitochondrial membrane transport systems highlighting mitochondrial carriers and enzymes reported to be modulated by suramin. Red arrows indicate targets strongly inhibited by suramin (K_i_ or IC_50_ lower than 50 µM), orange arrows indicate weak or partial inhibition, and the green hexagon denotes lack of detectable inhibitory activity. These interactions include high-affinity inhibition of ADP/ATP carriers (AACs), lower-affinity inhibition of aspartate/glutamate carriers (AGC1/2), weak inhibition of the ATP-Mg/Pi carrier (APC), no inhibition of the ornithine carrier (ORC1), and inhibition of succinate dehydrogenase (SDH) and carnitine O-acetyltransferase (CRAT). Abbreviations: MOM, mitochondrial outer membrane; MIM, mitochondrial inner membrane; IMS, intermembrane space; MM, mitochondrial matrix; AAC, ADP/ATP carrier; AGC, aspartate/glutamate carrier; APC, ATP-Mg/Pi carrier; ORC, ornithine carrier; SDH, succinate dehydrogenase; CRAT, carnitine O-acetyltransferase; TCA, tricarboxylic acid cycle; OXPHOS, oxidative phosphorylation.

Multiple independent studies have demonstrated that suramin binds and inhibits members of the mitochondrial SLC25 carrier family with a distinct but graded selectivity profile [17,19,82]. Among these carriers, the ADP/ATP carriers (AACs) represent the highest-affinity mitochondrial targets of suramin, among those tested, as evidenced by submicromolar inhibition constants measured in transport assays using recombinant human carriers reconstituted into proteoliposomes [17]. In particular, Todisco et al. (2016) [17] reported a K_i_ value of approximately 0.3 μM for suramin-mediated inhibition of human AAC2, establishing AACs as the most sensitive mitochondrial carriers to suramin (Table 2 and Figure 3). Comparative analyses across the SLC25 family revealed that suramin also inhibits the aspartate/glutamate carriers AGC1 and AGC2, albeit with lower affinity, with inhibitory constants in the low- to mid-micromolar range (K_i_ < 50 μM), as recently confirmed for AGC2 by transport assays, biophysical binding approaches, such as thermal stability shift assays, and ligand-induced stabilization analyses (Figure 3) [17,18]. In contrast, the ATP-Mg/Pi carrier (APC) exhibits only weak sensitivity to suramin, requiring higher micromolar concentrations for partial inhibition, while the ornithine carrier ORC1 is essentially insensitive under conditions that robustly inhibit AACs [17] (Table 2). Remarkably, in intact mitochondrial preparations and submitochondrial particles, Fujiwara et al. (2021) [83] observed dose-dependent inhibition of ADP uptake with IC_50_ values in the low-micromolar range (approximately 5 μM in intact mitochondria and 10 μM in submitochondrial particles) and demonstrated that suramin can exert its inhibitory effect from both the cytosolic and matrix-facing sides of the inner mitochondrial membrane. Together, these data indicate that suramin acts as a preferential inhibitor of AACs while retaining measurable, though weaker activity toward selected metabolite carriers such as AGC1 and AGC2. This graded selectivity within the SLC25 family suggests that suramin recognition is governed by carrier-specific electrostatic and structural features within the substrate-binding cavity rather than by a uniform mechanism shared across all mitochondrial carriers. From a mechanistic perspective, the differential inhibition observed among mitochondrial carriers may reflect the electrostatic complementarity between the highly polyanionic structure of suramin and the positively charged residues that line the substrate-binding cavity of several SLC25 transporters. In addition, variations in carrier-specific contact residues and gating elements that regulate access to the transport cavity may contribute to the observed selectivity. In this context, suramin binding could stabilize particular conformational states of the transporter and thereby interfere with the alternating-access mechanism that characterizes the transport cycle of mitochondrial carriers.

#### 3.3.2. Mitochondrial Enzymes

In addition to inner-membrane carriers, suramin has been reported to inhibit selected mitochondrial enzymes involved in oxidative metabolism, although the strength and translational relevance of evidence vary by target. Succinate dehydrogenase (SDH; complex II) is a historically proposed enzymatic target (Figure 3). Indeed, early enzymology studies reported inhibition of succinate oxidation by suramin and related polysulfonated compounds, with kinetic behavior interpreted as competitive with succinate, consistent with engagement of a dicarboxylate-recognition environment [16]. In line with mitochondrial metabolism being a sensitive node in clinically relevant systems, pathogen studies in kinetoplastids provide organism-level context indicating that suramin exposure remodels parasite metabolism and mitochondrial energy production (e.g., *Trypanosoma brucei* and *Crithidia fasciculata*) [84,85].

A newly reported mitochondrial enzyme target is represented by carnitine *O*-acetyltransferase (CRAT), a crucial enzyme involved in mitochondrial energy metabolism, considering that alterations in its activity have emerged as significant contributors to the pathogenesis of Leigh syndrome and related mitochondrial disorders [15,18,86]. CRAT catalyzes the reversible transfer of acetyl groups between acetyl-CoA and carnitine, thereby playing a key role in mitochondrial acetyl-CoA buffering and metabolic flexibility [84]. Cafferati Beltrame et al. (2025) [18] recently reported that suramin inhibits CRAT with IC_50_ values in the low- to mid-micromolar range by means of in silico docking analysis and by a combination of enzymatic assays, indicating that suramin can occupy the acetyl-CoA/carnitine binding region through electrostatic and steric complementarity (Figure 3).

A structurally well-supported interaction is suramin binding to sirtuin 5 (SIRT5), captured by X-ray crystallography of the SIRT5–suramin complex originally identified by biochemical screening using an NAD^+^-dependent deacetylase readout [12]. Because subsequent biochemical studies established dominant SIRT5 catalytic activities to be desuccinylation and demalonylation rather than deacetylation, at variance with what described for other structurally related SIRT homolog activities [87,88,89,90,91,92], the functional consequences of suramin binding should ideally be assessed using assays matched to these substrates. Accordingly, potency inferred from an NAD^+^-dependent deacetylase readout should not be overinterpreted. In this context, suramin is best presented as a validated ligand for SIRT5 with uncertain inhibitory potency across physiologically relevant deacylation reactions, pending direct biochemical confirmation.

Early biochemical observations, discussed in the context of carrier selectivity by Todisco et al. (2016) [17], also suggested that suramin is capable of targeting nucleotide- or anion-binding sites beyond membrane transporters, particularly within enzymes operating at the interface between the tricarboxylic acid cycle and oxidative phosphorylation. In this framework, succinate dehydrogenase (SDH, complex II of the respiratory chain), CRAT and SIRT5 emerged as a group of mitochondrial enzymes inhibited by suramin, consistent with its ability to engage positively charged regions involved in substrate (i.e., the adenine portion binding pocket of CoA in CRAT), or nucleotide cofactor (i.e., the adenine portion binding pocket of NAD^+^ in SIRT5 or FAD in SDH) recognition [84,85], depending on local electrostatic environments and structural accessibility.

Collectively, this evidence supports the view that suramin modulates mitochondrial metabolism through direct engagement of enzymatic components and alters oxidative stress and metabolic pathways, complementing its documented inhibition of selected SLC25 carriers and providing experimentally tractable entry points to dissect mitochondrial metabolic phenotypes across defined model systems.

**Table 2:** Mitochondrial targets of suramin. The exact numeric IC_50_/K_i_ values can be assay-dependent (proteoliposome vs. mitochondria vs. submitochondrial particles vs. TSA/ITC). The most robust primary values reported in the literature together with the described assays are reported. For precise curve-fit methods, substrate concentrations, liposome composition, and incubation times, please consult the primary articles cited in the table.

Target	Reported Potency (IC_50_/*K_i_*)	Assay Format/Conditions (Summary)	Primary Reference(s)
ADP/ATP carrier (human AAC2)	*K*_i_ ≈ 0.3 μM (competitive)	Recombinant AAC2 in proteoliposomes; radiolabeled ADP/ATP transport assays; docking + in vitro validation	[17]
ADP/ATP carrier (mitochondria)	IC_50_ ≈5 μM (mitochondria); ≈10 μM (submitochondrial particles)	[^3^H]ADP uptake into isolated mitochondria or submitochondrial particles; controls CATR and antimycin A	[83]
ADP/ATP carrier (human AAC1)	Low-μM range (~10 μM → ~90% inhibition)	Proteoliposome transport and thermal-shift (TSA) assays on recombinant AAC1	[93]
Aspartate/glutamate carrier (AGC2/SLC25A13)	Low–mid μM binding and transport inhibition	Docking → TSA → ITC → radiolabeled proteoliposome-based transport assays	[21]
Aspartate/glutamate carrier (AGC1)	Micromolar inhibition (lower potency than AAC2)	Proteoliposome-based transport assays and ligand selectivity analysis	[17]
Carnitine O-acetyltransferase (CRAT)	IC_50_ in low- to-mid-μM range	In vitro enzymatic activity assays (acetyl-CoA transfer measurement)	[18]
Succinate dehydrogenase (complex II)	Variable μM inhibition (across studies)	Spectrophotometric SDH enzyme assays (early enzymology and cellular metabolic tests)	[16,84]
ATP-Mg/Pi carrier (APC2)	Weak inhibition (relative to AAC2)	Proteoliposome-based transport assays; ligand selectivity analysis	[17]
Ornithine carrier (ORC1)	No detectable inhibition	Proteoliposome-based transport assays	[17]
SIRT5	IC_50_ in low- to mid-μM range	In vitro enzymatic activity assays	[12]

#### 3.3.3. Pharmacokinetic and Pharmacodynamic Considerations

The identification of mitochondrial enzymes and transporters as suramin targets suggests that the compound can influence cellular metabolism at multiple levels, linking its structural affinity for electropositive binding surfaces with broader effects on metabolic regulation. However, the interpretation of suramin interactions with intracellular and mitochondrial targets should be considered in the context of the compound’s distinctive pharmacokinetic properties. Suramin is a large, highly polyanionic molecule that does not readily cross biological membranes and is therefore administered intravenously in clinical settings. In plasma, the drug exhibits extremely strong binding to serum proteins and displays complex distribution kinetics characterized by a very long terminal half-life, which in humans may reach several weeks (approximately 40–50 days) [18,94,95,96]. This prolonged persistence largely reflects minimal metabolic transformation and slow renal elimination of the intact compound. These pharmacokinetic characteristics imply that the freely diffusible intracellular fraction of suramin may be substantially lower than the total circulating concentration, and therefore inhibitory effects observed in biochemical assays, often performed at micromolar concentrations, should be interpreted with appropriate caution when extrapolated to physiological conditions.

At the same time, the unusually long systemic exposure of suramin, together with its capacity to accumulate in certain tissues and to interact with electropositive protein surfaces, suggests that sustained low-level interactions with intracellular proteins cannot be excluded. In this context, mitochondrial enzymes and transporters may represent sensitive targets under conditions of prolonged exposure or local accumulation. Thus, while in vitro studies clearly demonstrate that suramin can interact with several mitochondrial proteins involved in energy metabolism, the pharmacological relevance of these interactions in vivo likely depends on exposure duration, tissue distribution, and cellular context. Consideration of these pharmacokinetic and pharmacodynamic aspects is therefore essential for interpreting both the therapeutic effects and the potential metabolic liabilities associated with this historically important drug.

Remarkably, recent studies exploring drug-delivery strategies have demonstrated that encapsulation of suramin into liposomal formulations can enhance cellular uptake and improve intracellular antiviral activity, highlighting the potential of nanocarrier-based delivery systems to overcome the intrinsic membrane-permeability limitations of this highly charged molecule [97,98].

### 3.4. Structurally Characterized Targets of Suramin

In this section, we focus specifically on proteins for which crystallographic structures of suramin–protein complexes have been reported, providing direct structural evidence of ligand binding. Beyond mitochondrial carriers and enzymes, a growing number of suramin–protein interactions have been elucidated at the structural level, providing direct molecular insight into binding modes across diverse biological systems. These structures complement functional and biochemical studies discussed in previous sections and collectively reinforce the view of suramin as a polyvalent ligand that preferentially engages extended basic surfaces and nucleotide- or anion-binding regions. To avoid redundancy, only protein families not extensively discussed elsewhere in the manuscript are briefly highlighted here, with particular emphasis on the implications of sequence conservation for target extrapolation.

Several crystallographic studies have captured suramin bound to viral proteins, including RNA-dependent RNA polymerases (i.e., 7yet.pdb [13] or 3ur0.pdb [97], Figure 2A,B and Table S1) and nucleocapsid proteins (i.e., 4j4v.pdb [99], Figure 2C and Table S1) from RNA viruses. While the antiviral activity of suramin has been presented in Section 3.1, the availability of these structures provides a molecular basis for its broad antiviral profile. Notably, the high sequence identity among homologous viral polymerases and nucleocapsid proteins (often exceeding 70–90%) suggests that suramin binding observed in these systems may extend to closely related viral targets, supporting the relevance of these structures beyond the specific species crystallized (Figure 2 and Table S1). Remarkably, the BLASTp analysis reported in Supplementary Table S1 is intended primarily as a hypothesis-generating approach to identify structurally related proteins that may share electrostatic or structural features potentially compatible with suramin binding. However, sequence similarity alone does not necessarily imply conservation of ligand-binding residues, and therefore structural or biochemical validation remains necessary to confirm potential suramin–protein interactions. Structural data also reveal that trypanosome strains that express the variant surface glycoprotein (VSG) VSGsur possess heightened resistance to suramin (i.e., 4j4v.pdb [100], Figure 2D and Table S1). Although the antiparasitic activity of suramin is discussed in detail elsewhere, these complexes further corroborate its ability to bind conserved folds involved in host–parasite interactions and parasite metabolism. High sequence similarity among homologous parasitic proteins supports the notion that suramin may act on multiple members of related protein families through shared electrostatic recognition principles (Figure 2B).

Among non-parasitic and non-viral targets, several enzymes and regulatory proteins have been structurally characterized in complex with suramin. These include proteins structurally related to the phospholipase A2 (PLA2) family, with reference to those proteins derived from snake venom myotoxins (i.e., 6ce2.pdb [101], 1y4l.pdb [36], 4yv5.pdb [102], 3bjw.pdb [103], Figure 2E and Table S1). Although these enzymes are evolutionarily distant from human PLA2s, BLASTp analysis reveals moderate to high sequence identity (approximately 45–55%, Table S1) with mammalian homologs. Structural data indicate that suramin binds to extended basic regions proximal to the catalytic groove, suggesting a potential, albeit context-dependent ability to interfere with phospholipid-binding surfaces. This observation is consistent with suramin’s reported anti-inflammatory properties, while not implying direct inhibition of endogenous PLA2 isoforms (Figure 2E).

Suramin has also been crystallized in complex with chromobox (CBX) family chromodomains, including CBX4, CBX7, and CBX8, which are components of polycomb repressive complexes involved in chromatin regulation (i.e., 4x3u.pdb [104], Figure 2F and Table S1). These chromodomains display high sequence identity with their human counterparts (often >70–90%), and structural analyses show suramin occupying positively charged regions implicated in histone tail recognition. While functional modulation of CBX proteins by suramin has not been systematically explored, these structures provide a plausible molecular framework linking suramin binding to previously reported effects on transcriptional and cancer-related pathways (Figure 2F).

Additional structural targets include nuclear transcription factor Y (NF-Y) subunits, which form a highly conserved heterotrimeric complex that binds CCAAT promoter elements. Suramin-bound NF-Y proteins exhibit strong sequence conservation across species, including near-complete identity with human NF-Y subunits. Although direct functional inhibition has not yet been demonstrated, the structural data suggest that suramin may interfere with protein–protein or protein–DNA interfaces through electrostatic masking, further expanding its potential nuclear interactome (i.e., 7ah8.pdb [105], Figure 2G and Table S1).

Suramin has also been structurally characterized in complex with sirtuin 5 (SIRT5), a member of the NAD^+^-dependent sirtuin family primarily localized to mitochondria (i.e., 2nyr.pdb [12] Table S1 and Figure 2H). In the crystal structure, suramin occupies a positively charged region overlapping the NAD^+^-binding site, consistent with its ability to engage nucleotide-recognition pockets. The high sequence conservation of SIRT5 across species and the availability of a suramin-bound structure support its classification as a validated structural interactor while underscoring the need for activity-matched biochemical assays to define its physiological sensitivity to suramin, also compared to other members of sirtuins [87,88,89,90,91,92].

A structurally well-defined interaction has also been reported for suramin binding to an E3 ubiquitin ligase domain, exemplified by the PRY/SPRY domain of TRIM21 (i.e., 9gte.pdb [66], Table S1 and Figure 2I). The crystal structure reveals suramin engaging a basic surface involved in protein–protein recognition rather than the catalytic ubiquitin transfer site. Given the high sequence similarity between the crystallized construct and the human TRIM21 protein, this interaction provides a molecular basis for suramin’s reported modulation of ubiquitin-dependent signaling pathways. More broadly, it suggests that suramin may interfere with regulatory E3 ligases by masking interaction interfaces, rather than through classical enzyme inhibition.

Suramin has additionally been crystallized in complex with thrombin, a serine protease central to coagulation and inflammatory signaling (i.e., 2h9t.pdb and 3bf6.pdb [9], Table S1 and Figure 2J). Structural analyses show suramin binding to extended basic exosites rather than the catalytic triad, consistent with an allosteric mode of interaction that may influence substrate or cofactor recognition. Given the high conservation of thrombin among vertebrates, these structures provide a mechanistic rationale for the reported anticoagulant and anti-inflammatory effects of suramin while highlighting its preference for electropositive surface regions involved in macromolecular assembly rather than active-site blockade.

In parasitic systems, suramin has been structurally characterized in complex with pyruvate kinase from *Leishmania* species (i.e., 3pp7.pdb [10], Figure 2K and Table S1), a key glycolytic enzyme essential for parasite energy metabolism. The structure reveals suramin bound at a positively charged surface proximal to the nucleotide-binding region, consistent with inhibition of phosphotransfer reactions. While parasite pyruvate kinases display lower sequence identity to human isoforms, conservation of core catalytic architecture supports the relevance of this interaction for antiparasitic activity. These data reinforce the concept that suramin can target central metabolic enzymes in kinetoplastids through conserved electrostatic recognition principles.

Finally, suramin has been crystallized with a limited number of proteins of uncertain or non-human biological relevance, including plant proteins (i.e., 3gan.pdb, Figure 2L and Table S1) involved in nucleic acid–related processes. While these targets are not directly pertinent to human pharmacology, they underscore suramin’s propensity to bind conserved structural motifs, particularly in proteins enriched in basic residues or nucleic acid-binding domains. Such structures reinforce the predictive value of sequence similarity, especially when identity exceeds ~40%, in anticipating additional suramin-interacting proteins across species.

From a structural perspective, the available crystallographic complexes consistently show suramin occupying extended electropositive grooves or nucleotide-binding pockets. This recurring binding mode supports the idea that electrostatic multivalency represents a key determinant of suramin’s ability to interact with diverse protein families. Collectively, the structurally characterized non-mitochondrial targets of suramin illustrate how direct binding observed in crystallographic complexes can inform broader target landscapes through sequence conservation. When integrated with biochemical and cellular evidence, these data emphasize the polypharmacological nature of suramin and provide a structural foundation for understanding both its therapeutic potential and its context-dependent biological effects (Table S1 and Figure 2). Taken together, the expanding catalog of structurally and biochemically validated suramin targets provides a framework for predictive target discovery. In this context, the well-documented cross-reactivity of suramin, often regarded as a limitation in target selectivity, can be used as an advantage, as conserved structural and electrostatic features among homologous proteins enable the rational identification of suramin-responsive targets across diverse microorganisms, including protists, fungi, bacteria, and viruses, using genomics-guided and structure-based approaches [20,106,107,108,109,110]. Such approaches enable the prioritization of candidate suramin-sensitive proteins even in organisms with limited experimental characterization, thereby extending the translational relevance of existing structural data.

### 3.5. Antiparasitic Activity and Parasite-Specific Targets

Suramin remains a cornerstone therapy for the early stage of African trypanosomiasis and exerts its antiparasitic activity through a multifactorial mechanism. In trypanosomes, suramin is internalized via receptor-mediated endocytosis involving the invariant surface glycoprotein ISG75 and a lysosome-associated major facilitator superfamily transporter. Once internalized, suramin has been shown to inhibit multiple parasite-specific enzymes, including glycolytic enzymes such as hexokinase, aldolase, phosphoglycerate kinase, and glycerol-3-phosphate dehydrogenase. These enzymes are localized within glycosomes, and the precise route by which suramin accesses them remains incompletely understood [111,112].

Additional parasite targets proposed to contribute to suramin’s trypanocidal activity include glycerophosphate oxidase, the serine oligopeptidase OP-Tb, and the RNA-editing ligase REL1 of the kinetoplast. Although suramin is generally considered poorly permeable to biological membranes, inhibition of oxidative phosphorylation has been demonstrated in mitochondrial preparations from *Crithidia fasciculata*, supporting its capacity to interfere with mitochondrial energy metabolism in trypanosomatids [111,113] and cross the mitochondrial membrane.

Furthermore, suramin disrupts cytokinesis in *Trypanosoma brucei*, as evidenced by the accumulation of binucleated parasites following treatment. Clinically, suramin is administered intravenously for the treatment of early-stage *T. b. rhodesiense* infection and as a second-line option for *T. b. gambiense*. While effective, treatment is associated with adverse effects including renal dysfunction, peripheral neuropathy, hypersensitivity reactions, and bone marrow toxicity [111,113,114]. Drug resistance has been linked to loss-of-function mutations in transporters involved in suramin uptake, underscoring the importance of parasite-specific internalization mechanisms [111,115].

Recent evidence also suggests that divalent cations such as Mg^2+^, Ca^2+^, and Zn^2+^ may modulate suramin’s antiparasitic activity, adding an additional layer of complexity to its mode of action (as suggested by Hauser et al. 2023 [116]). Structural support for this interaction has been provided by crystallographic studies, which captured suramin bound to variant surface glycoproteins of *Trypanosoma brucei*, further corroborating the role of ISG/VSG family members in suramin recognition and internalization (see Table S1 and Figure 2).

## 4. Analogues of Suramin and Structure-Activity Relationships

Several analogues of suramin have been designed [117] and/or studied as antagonists of purinergic receptors [29,118] and SIRT receptors [119].

**Figure 4:**
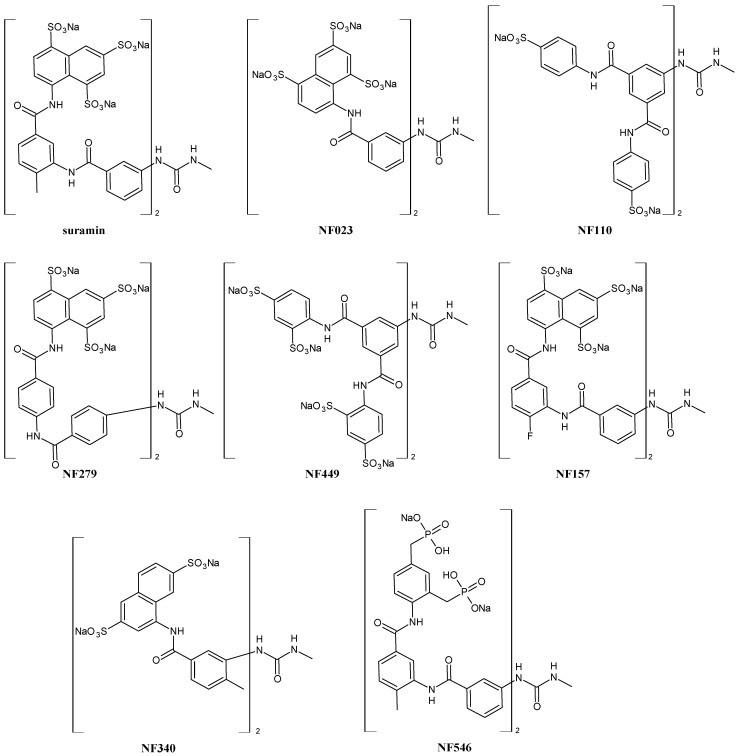
Structures of suramin and its analogues **NF023**, **NF110**, **NF279**, **NF449**, **NF157**, **NF340**, and **NF546**.

The most common analogues named with the acronym NF are summarized in Figure 4. The first antagonist of suramin studied was **NF023** (8,8′-carbonylbis[imino-3,1-phenylencarbonylimino-(3,1-phenylen)carbonylimino]-bis-1,3,5-naphthalentrisulfonic acid), a competitive and reversible antagonist at P2X1 receptors. It can be considered a simplified analogue of suramin, obtained by formal removal of an aminotoluic moiety. It is also selective for P2X1 receptors over P2X2, P2X3, P2X4, and P2Y receptors and is a weak inhibitor of ATP metabolism by ectonucleotidases.

**NF110** [4,4′,4″,4‴-(carbonylbis(imino-5,1,3-benzenetriylbis(carbonylimino)))tetrakis-benzenesulfonic acid] is a more potent P2X1 antagonist than **NF023**, with a *K*_i_ of 82 nM, but is even more potent at P2X3 receptors [120]. It is a lower benzologue of both suramin and **NF023**, symmetrically decorated with an additional benzanilido substituent. It also inhibits IP5K to levels comparable with suramin [40].

**NF279**, 8,8′-(carbonylbis(imino-4,1-phenylenecarbonylimino-4,1-phenylenecarbonylimino))bis(1,3,5-naphthalenetrisulfonic acid) is an important agonist of suramin. It may be considered as a regioisomer of norsuramin (it differs from suramin in the position of both benzamido groups linked to the diarylurea and lacks the methyls). It was first disclosed in 1998 by Damer et al. [121] who demonstrated that this compound antagonized P2X receptor-mediated contractions in rat vas deferens, evoked by *α*,*β*-methylene ATP (10 μM; *p*IC_50_ = 5.71) without affecting responses mediated via *α*_1A_-adrenoceptors, adenosine A_1_ and A_2B_ receptors, histamine H_1_, muscarinic M_3,_ and nicotinic receptors. The compound also showed low inhibitory potency against P2Y receptors in guinea-pig taenia coli and at ecto-nucleotidases in folliculated *Xenopus laevis* oocytes (IC_50_ > 100 μM), indicating this compound as a specific and selective P2X receptor antagonist. Till then, several studies were addressed to this compound. In 2000, Rettinger et al. [122] analysed the P2X receptor subtype selectivity, demonstrating that it is a potent P2X1 receptor-selective and reversible antagonist. It is a more potent antagonist at P2X1 receptors than suramin or **NF023**, being active in the subnanomolar range [28], and is selective for P2X1 receptors over P2X2, P2X3, P2X4, P2X7, and P2Y receptors (P2X1 > P2X2 ≥ P2X3 ≥ P2X7 >> P2X4) [123]. **NF279** is also a weak inhibitor of ATP breakdown by ectonucleotidases. In the study by Chanalaris et al. (2017) [62], porcine cartilage explants were treated with retinoic acid (1 mM) and/or suramin analogues (200 µg/mL) for 48 h. Compound **NF279** showed markedly higher activity than suramin, inhibiting both retinoic acid–stimulated and unstimulated release of aggrecan fragments, with an IC_50_ of 15.6 ± 10 µg/mL compared to that of suramin (IC_50_ = 98 ± 9 µg/mL).

**NF449** [4,4′,4″,4‴-(carbonylbis(imino-5,1,3-benzenetriylbis(carbonylimino)))tetrakis-benzene-1,3-disulfonic acid] was a highly potent P2X1 receptor antagonist at recombinant rat P2X1 receptors, being even most potent than **NF279** [124]. It was also more potent than **NF110**, from which it differs structurally only by having two instead of one sulfate salt residues per benzene ring. Moreover, the selectivity between P2X1 and P2X7 receptor subtypes was studied by Hülsmann et al. (2003) [125], as demonstrated against homomeric human P2X1 and P2X7 receptor subtypes (hP2X1 and hP2X7) heterologously expressed in *Xenopus oocytes* by means of the two-microelectrode voltage-clamp technique. It was demonstrated that compound **NF449** was able to discriminate between both receptors in native human tissues, acting as a reversible competitive antagonist at the hP2X1 with much higher potency at hP2X1 than at hP2X7 receptors. Zhang et al. (2020) [40] demonstrated that **NF449**, as well as suramin, suppresses IP6 production by targeting IP5K, inhibiting ATP-binding activity and IP5 phosphorylation. NF449 is a more potent IP5K inhibitor than suramin, with its apparent IC_50_ (1.1 μM) ∼3-fold lower than that of suramin.

Compound **NF157**, 8,8′-[carbonylbis[imino-3,1-phenylenecarbonylimino(4-fluoro-3,1-phenylene) carbonylimino]]bis-1,3,5-naphthalenetrisulfonic acid hexasodium salt, may be considered a bioisoster of suramin since it bears two fluorine atoms replacing suramin’s methyl groups. It was the first nanomolar P2Y11 antagonist (**NF157**, p*K*_i_ = 7.35 ± 0.06 against P2Y11 receptors recombinantly expressed in 1321N1 astrocytoma cells [29]). The p*K*_i_ value reported for suramin was 6.52  ±  0.13. Compound **NF157** also displayed selectivity for P2Y11 over P2Y1 and P2Y2 (>650-fold), P2X7 (>67-fold), P2X4 (>22-fold), P2X3 (8-fold) and P2X2 (3-fold) but no selectivity over P2X1.

**NF340**, 4,4′-(carbonylbis(imino-3,1-[4-methyl-phenylene]carbonylimino))bis(naphthalene-2,6-disulfonic acid), inhibits the activity of P2Y11 receptor by completely combining with ATP-binding amino acid residues. This toluoyl derivative lacks the benzamide connecting moiety and one sulfate group on the naphthalene ring. **NF340** is known to display 520-fold higher selectivity for P2Y11 than other P2Y family receptors [126,127]. It had four times as much antagonistic potency as **NF157** in a Ca^2+^-based assay and ten times the potency in a cAMP assay. This compound displayed P2Y11 selectivity over P2Y1,2,4,6,12 and P2X1,2,2-3 [128]. Green et al. (2023) [129] recently evaluated the protective activity of cartilage exerted by some suramin analogues against osteoarthritic breakdown by increasing levels of TIMP3 in the tissue compared to suramin. *K*_d_ values for binding to TIMP3 were determined by ELISA and compared with suramin (*K*_d_ = 1.90 ± 0.21 nM). Analogue **NF279** (*K*_d_ = 0.85 ± 0.09 nM) was the most potent, followed by **NF157** (*K*_d_ = 1.55 ± 0.11 nM).

The selective non-nucleotide P2Y11 agonist **NF546** [4,4′-(carbonylbis(imino-3,1-phenylene-carbonylimino-3,1-(4-methyl-phenylene)carbonylimino))bis(1,3-xylene-α,α′-diphosphonic acid) tetrasodium salt] was identified by Meis et al. (2010) [126]. This phosphonic bioisoster had a *p*EC_50_ of 6.27 and is relatively selective for P2Y11 over P2Y1, P2Y2, P2Y4, P2Y6, P2Y12, P2X1, P2X2, and P2X2-X3. **NF546** was further tested for P2Y11-mediated effects in monocyte-derived dendritic cells, demonstrating that it led to thrombospondin-1 secretion and inhibition of lipopolysaccharide-stimulated interleukin-12 release, whereas NF340 inhibited these effects. Moreover, it was shown that **NF546** stimulation promotes interleukin 8 (IL-8) release from dendritic cells, which could be inhibited by **NF340**.

Parveen et al. (2022) [130] reported the synthesis of several suramin derivatives acting as antiproliferative agents via FGF1 and FGFRD2 blockade, suggesting some of them (**14**, **15**, **17**, **18**, and **21**) as better antimetastatic agents than suramin, also being less toxic than suramin (Table 3). The IC_50_ values of the synthesized compounds in MCF-7 breast cancer cells were analyzed using SigmaPlot software (version 12.5, Systat Software Inc., San Jose, CA, USA). The estimated IC_50_ values in MCF-7 cells are reported in Table 3 and compared to suramin (IC_50_ = 153.96 ± 1.44 μM).

Recently, two analogues of suramin previously synthesized (NF031 or **RT5**, and NF032 or **RT14,** see Table 3) and two new analogues (**RT31** and **RT58**) were studied for their antitrypanocidal activity, demonstrating the importance of the methyl group on the intermediate rings and the specific regiochemistry of naphthalenetrisulfonic acid groups, or sodium salt groups [131]. These compounds differ from suramin also in the position of a sulfate group on the naphthalene group. Specifically, the two 1,3,5-naphthalenetrisulfonic acid groups were replaced with two 1,3,6-naphthalenetrisulfonic acid groups, and the regiochemistry of the phenyl rings was analyzed (**RT5** (NF031): *para*/*para*; **RT14** (NF032): *meta*/*para*; **RT31**: *para*/*meta*; **RT58**: *meta*/*meta* like suramin). Compounds **RT5** (NF031), **RT31**, and **RT58** suppressed the proliferation of bloodstream forms of *T. brucei*; **RT14** (NF032) affected the growth of the parasite only marginally. However, **RT5** (NF031), **RT31,** and **RT58** were 1000 times (minimum inhibitory concentration, MIC values) and more than 500 times (50% growth inhibition, GI_50_ values), respectively, less potent in inhibiting the growth of bloodstream-form trypanosomes than suramin (MIC = 0.1 µM, GI_50_ = 0.04 ± 0.01 µM). The authors conclude that the methyl groups on the intermediate rings and the regiochemistry of the naphthalenetrisulfonic acid groups are important structural features for the superior trypanocidal activity of suramin, and suggest the study of more suramin analogues with different regiochemistry of the naphthalenetrisulfonic acid groups and with or without methyl groups on the intermediate rings to confirm this hypothesis.

In the study by Pillaiyar et al. (2020) [132] (Table 3), the synthesis and biological evaluation of several small molecules, which are suramin-derived dual antagonists for the proinflammatory G protein-coupled receptors P2Y2 and the orphan receptor GPR17, were reported (**14b**, **14m**, and **14l**, Table 3). These compounds were suggested as interesting starting points for developing potent multi-target drugs with potential applications in inflammatory diseases. These compounds were also reported recently in a perspective by Puthanveedu et al. (2025) [131].

Viewed collectively, the diverse biological interactions reported for suramin can be interpreted within a unified framework in which electrostatic multivalency enables promiscuous recognition of nucleotide- and anion-binding protein surfaces, a feature that is preserved to varying degrees across many of its synthetic analogues.

**Table 3:** Analogues of suramin reported by Parveen et al. (2022) [130] and Steverding et al. (2024) [133] and small-molecule analogues reported by Pillaiyar et al. (2020) [132].

Structure	Cmpd	K_d_ and Activity	Reference
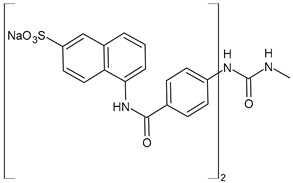	**14**	*K*_d_ = 13.70 μM (FGF1) Estimated IC_50_ = 408.8 ± 3.08 μM (MCF-7)	Parveen et al. (2022) [130]
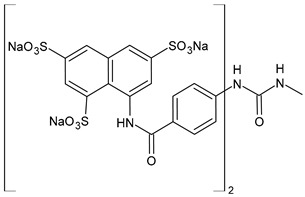	**15**	*K*_d_ = 20.9 μM (FGF1) Estimated IC_50_ = 193.04 ± 1.57 μM (MCF-7)	Parveen et al. (2022) [130]
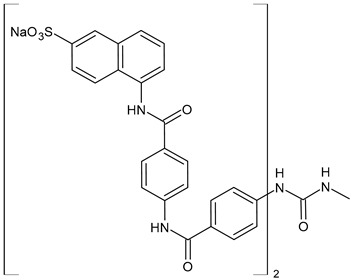	**17**	*K*_d_ = 10.1 μM (FGF1) Estimated IC_50_ = 619.4 ± 1.73 μM (MCF-7)	Parveen et al. (2022) [130]
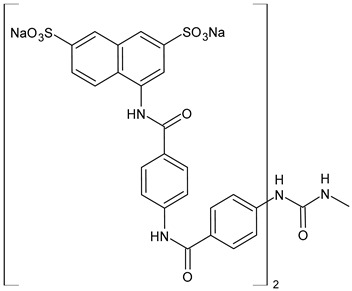	**18**	*K*_d_ = 15.50 μM (FGF1) Estimated IC_50_ = 325.63 ± 2.03 μM (MCF-7)	Parveen et al. (2022) [130]
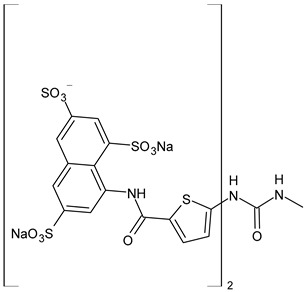	**21**	*K*_d_ = 15.9 μM (FGF1) Estimated IC_50_ = 417.01 ± 0.99 μM (MCF-7)	Parveen et al. (2022) [130]
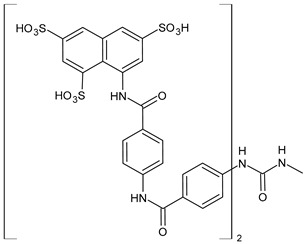	**RT5 (NF031)**	MIC = 100 µM (*T. brucei*) GI_50_ = 21.7 ± 4.0 µM (*T. brucei*)	Steverding et al. (2024) [133]
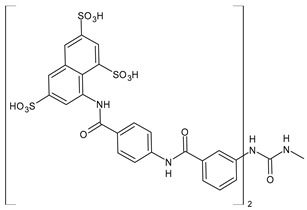	**RT14 (NF032)**	MIC > 100 µM (*T. brucei*) GI_50_ > 100 µM (*T. brucei*)	Steverding et al. (2024) [133]
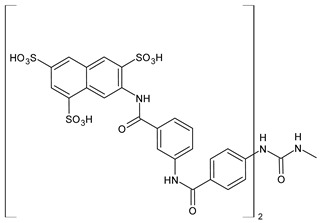	**RT31**	MIC = 100 µM (*T. brucei*) GI_50_ = 30.5 ± 3.3 µM (*T. brucei*)	Steverding et al. (2024) [133]
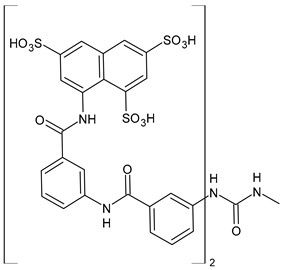	**RT58**	MIC = 100 µM (*T. brucei*) GI_50_ = 29.9 ± 1.7 µM (*T. brucei*)	Steverding et al. (2024) [133]
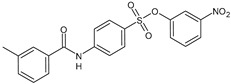	**14b**	IC_50_ = 3.43 ± 0.68 μM (P2Y_2_R) IC_50_ = 1.92 ± 0.30 μM (GPR17)	Pillaiyar et al. (2020) [132]
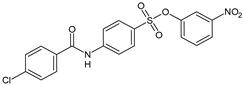	**14l**	IC_50_ = 3.01 ± 0.54 μM (P2Y_2_R) IC_50_ = 3.37 ± 0.66 μM (GPR17)	Pillaiyar et al. (2020) [132]
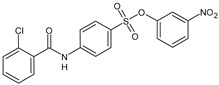	**14m**	IC_50_ = 3.17 ± 0.83 μM (P2Y_2_R) IC_50_ = 1.67 ± 0.16 μM (GPR17)	Pillaiyar et al. (2020) [132]

## 5. Limitations, Polypharmacology, and Future Directions

Despite sustained clinical and preclinical interest, the translation of suramin beyond its established antiparasitic indications remains constrained by well-recognized pharmacokinetic and safety liabilities. In oncology development, for example, complex pharmacokinetics (including a prolonged terminal half-life) and dose-limiting neurotoxicity have been recurrent practical constraints, motivating careful exposure targeting and renewed interest in delivery strategies and analogue design aimed at mitigating systemic exposure [2,134]. Against this background, local delivery approaches represent a rational future direction to harness suramin bioactivity while minimizing systemic exposure. In this context, the activity of suramin against epithelial ulcers has been recently addressed. In a recent study by Encinas-Basurto et al. (2025) [135], carboxymethyl chitosan–based hydrogels were cross-linked with genipin and evaluated for the controlled release and tissue retention of suramin. It was demonstrated that the hydrogel based on carboxymethyl chitosan cross-linked with genipin can improve the local delivery of suramin by modulating its release and retention through controlled cross-linking density, thus enhancing its therapeutic potential for epithelial ulcers (e.g., in the investigated epithelial ulcer model HSV-1 keratitis–linked ulceration [135]). Despite known adverse effects—particularly at higher exposures—several clinical studies have assessed suramin in autism spectrum disorder (ASD), cancer, and other disease contexts, as summarized in Table 4. Naviaux et al. (2017) [22] reported the results of the Suramin Autism Treatment-1 (SAT-1) trial (NCT02508259), a double-blind, placebo-controlled, translational pilot study that studied the safety and activity of low-dose suramin in children (10 males with ASD, ages 5–14 years) with ASD. No serious side effects were observed; there was only a self-limited, asymptomatic rash. Secondary outcomes also showed improvements in language, social interaction, and decreased restricted or repetitive behaviors. Wu et al. (2023) [95] reported the results of a double-blinded, randomized, placebo-controlled single ascending dose study (NCT03804749) investigating the pharmacokinetics, tolerability, and safety of suramin in 36 healthy Chinese volunteers aged 18–45 years. Suramin was generally well tolerated. Doses of 10 mg/kg or 15 mg/kg were suggested as appropriate doses for multiple-dose studies. Hough et al. (2023) [136] reported the findings of a 14-week, randomized, double-blind, placebo-controlled proof-of-concept study (NCT06058962) to test the efficacy and safety of suramin intravenous infusions in children aged 4–15 years with moderate to severe ASD. Suramin was generally safe and well tolerated over 14 weeks; most adverse events were mild to moderate in severity. Finally, a randomized, double-blind, crossover, 30-week clinical trial (STAT-2A; NCT06866275) is underway to evaluate the preliminary proof of concept, safety, and pharmacokinetics of suramin sodium (KZ101) with repeat dosing by intravenous infusion in males 5–14 years of age who have been diagnosed with ASD.

Several clinical trials evaluated the use of suramin in cancer, specifically non-small-cell lung cancer (NSCLC), hormone-refractory prostate cancer (HRPC), and adrenocortical cancer (NCT 00002921), historically motivated by suramin’s ability to interfere with growth factor signaling and other extracellular interactions [137]. However, oncology experience has been shaped by exposure management, toxicity (including neuropathy), and variable efficacy, and is best discussed with explicit attention to dosing strategy, pharmacokinetic targets, and endpoints rather than as uniformly positive activity. A phase III clinical study (NCT00002723) regarding prostate cancer was reported by Vogelzang et al. in 2004 [138]. In this study, prostate-specific antigen (PSA) and objective response rates associated with suramin administration were evaluated in patients with hormone-refractory prostate carcinoma (HRPC). A monthly × 3 one-hour infusion bolus of suramin was well tolerated, reduced PSA levels, and induced objective responses, even in patients who previously had received chemotherapy. Representative trials in NSCLC and metastatic prostate cancer illustrate both the rationale and the practical constraints that have limited broad adoption [139,140,141]. From a translational perspective, actionable next steps include (i) localized delivery strategies to reduce systemic exposure and (ii) structure-guided optimization of suramin-derived chemotypes to improve pharmacokinetic behavior. In parallel, integrating target-engagement biomarkers with well-controlled functional assays will be essential to deconvolute suramin’s polypharmacology and to prioritize the molecular interactions most relevant for therapeutic repurposing.

**Table 4:** Clinical studies involving the administration of suramin.

Name	Number [142]	Phase	Status
University of California, San Diego (UCSD) Suramin Autism Treatment-1 (SAT1) Trial (SAT1)	NCT02508259	Interventional study Phase I/II	Completed
Study to Evaluate the Tolerance and Pharmacokinetics of Suramin Sodium	NCT03804749	Interventional study Phase I	Unknown status
Double Blind Trial in Children with Autism Spectrum Disorder	NCT06058962	Interventional study Phase II	Completed
Suramin for the Treatment of Autism Trial: KZ101 in a Male Pediatric Population with Autism Spectrum Disorder (ASD)	NCT06866275	Interventional study Phase II	Active-Recruiting
Evaluation of Non-cytotoxic Suramin as a Chemosensitizer in Non-small Cell Lung Cancer	NCT01038752	Interventional study Phase II	Terminated
Low, Intermediate, or High Dose Suramin in Treating Patients with Hormone-Refractory Prostate Cancer	NCT00002723	Interventional study Phase III	Completed
S9427, Suramin in Treating Patients with Stage III or Stage IV Adrenocortical Cancer Incurable by Surgery	NCT 00002921	Interventional study Phase II	Terminated

## 6. Discussion

The body of evidence summarized in this review highlights suramin as an unusually persistent pharmacological entity whose biological relevance has not diminished with time, but rather expanded as experimental approaches have evolved. Historically framed as an extracellular antagonist of purinergic signaling and growth factor interactions, suramin is now clearly recognized as a compound capable of engaging a broad range of intracellular and mitochondrial targets, including metabolite carriers and enzymes central to cellular energy metabolism. This expanded target spectrum provides a unifying framework to interpret both the therapeutic effects and the dose-limiting toxicities that have shaped suramin’s clinical history [1,2].

A recurring theme emerging from biochemical, structural, and cellular studies is that suramin’s polypharmacology is not accidental, but rooted in its highly polyanionic architecture [1,2,3]. Electrostatic complementarity between sulfonate groups and extended basic cavities enables suramin to bind nucleotide- and anion-recognition sites across diverse protein families, from purinergic receptors to SLC25 mitochondrial carriers and metabolic enzymes [17,18,19,20,21]. While this feature underlies the compound’s sub- to low-micromolar potencies across multiple targets, it also explains its limited selectivity and the difficulty of uncoupling beneficial effects from systemic liabilities. In this respect, suramin exemplifies the trade-off between potency and specificity that characterizes many early pharmacological scaffolds.

The identification of mitochondrial carriers, including AAC, AGC, and APC, as suramin targets is particularly significant from a conceptual standpoint. These findings challenge the long-standing assumption that inner-mitochondrial membrane transporters are largely inaccessible to small-molecule modulation and instead position them as viable pharmacological targets [17,20,21]. At the same time, the engagement of mitochondrial enzymes such as SDH, CRAT, and SIRT5 suggests that suramin can influence metabolic flux and redox balance at multiple levels [12,15,16,18]. Such multilayered mitochondrial perturbation likely contributes to the compound’s documented effects in both host and pathogen systems, while also providing a plausible mechanistic basis for adverse outcomes observed at higher or prolonged exposures.

Clinical experience with suramin further reinforces this duality. However, many of the therapeutic indications proposed for suramin originate primarily from exploratory or preclinical studies, and therefore their translational relevance should be interpreted cautiously. In oncology, enthusiasm driven by interference with extracellular growth factor signaling has been tempered by complex pharmacokinetics, prolonged persistence in vivo, and dose-limiting toxicities, including neuropathy [2,132,136,137,138,139,140]. These pharmacokinetic properties, including the unusually long systemic half-life of suramin, have historically complicated dose optimization and contributed to the narrow therapeutic window observed in several clinical studies. In contrast, more recent low-dose clinical studies in autism spectrum disorder have demonstrated that carefully controlled exposure can achieve measurable biological effects with acceptable tolerability [22,95,135]. These observations underscore the importance of exposure management and target prioritization when considering suramin beyond its established antiparasitic use. Consequently, the potential repurposing of suramin in new therapeutic contexts requires careful evaluation of the balance between pharmacological efficacy and systemic toxicity.

From a translational perspective, the most promising future directions lie not in broad systemic repurposing, but in strategies that exploit suramin’s biology while mitigating its liabilities. Local delivery approaches, such as biomaterial-based depots for epithelial ulcers, illustrate how spatial control of exposure can enhance therapeutic potential while limiting systemic accumulation [134]. In parallel, structure-guided optimization of suramin-derived chemotypes offers a rational path toward improved selectivity, pharmacokinetics, and tissue distribution. Importantly, advances in structural biology, genomics, and target identification now make it feasible to systematically explore cross-reactivity within conserved protein families, particularly in pathogens, turning a historical limitation of suramin into a potential advantage.

Taken together, these considerations position suramin less as a conventional drug candidate and more as a reference scaffold and chemical probe. Its ability to reveal vulnerabilities in mitochondrial transport, metabolic regulation, and host–pathogen interactions exemplifies how revisiting legacy compounds with modern tools can generate insights of both mechanistic and translational value. More generally, future research should focus on identifying the most pharmacologically relevant targets and on developing optimized derivatives or delivery strategies capable of improving selectivity, intracellular exposure, and overall therapeutic safety.

## 7. Conclusions

Suramin represents a rare example of a century-old drug that continues to reveal new molecular mechanisms. Once regarded primarily as an extracellular antagonist of purinergic signaling, suramin is now recognized as a broad-spectrum modulator of intracellular and mitochondrial targets, including multiple members of the SLC25 carrier family and key metabolic enzymes. These findings significantly expand our understanding of suramin’s pharmacological profile and help rationalize both its therapeutic efficacy and its adverse effects.

From a mechanistic perspective, suramin’s polyanionic structure enables high-affinity interactions with nucleotide- and metabolite-binding proteins, particularly those enriched in basic residues. While this feature underlies its remarkable potency, it also explains its limited selectivity and unfavorable pharmacokinetic properties. Importantly, the identification of mitochondrial carriers as suramin targets challenges the long-standing view that these proteins are pharmacologically inaccessible and opens new avenues for drug discovery.

Future research should aim to disentangle beneficial from deleterious effects by developing suramin-derived or -inspired molecules with improved selectivity and reduced systemic persistence. In this context, suramin should be viewed not only as a therapeutic agent but also as a valuable chemical probe for exploring mitochondrial transport, metabolic regulation, and host–pathogen interactions. The continued study of this compound exemplifies how revisiting legacy drugs with modern tools can yield insights of both fundamental and translational relevance.

## Figures and Tables

**Table 1:** Extra-mitochondrial molecular targets of suramin.

Target	Cellular Location	Reported Potency	Assay/Model	Reference
P2X/P2Y receptors	Plasma membrane	IC_50_ ~1–10 μM	Electrophysiology, Ca^2+^ flux	[2]
Na^+^/K^+^-ATPase	Plasma membrane	μM range	ATP hydrolysis assay	[2]
Pyruvate kinase	Cytosol	μM range	Enzyme kinetics	[2]
DNA/RNA polymerases	Nuclear-/-viral	μM range	In vitro polymerase assays	[2]
Growth factor binding	Extracellular	Qualitative	Ligand displacement	[2]

## Data Availability

The original contributions presented in this study are included in the article/Supplementary Materials. Further inquiries can be directed to the corresponding author.

## Supplementary Materials

The following supporting information can be downloaded at https://www.mdpi.com/article/10.3390/biom16040527/s1, Table S1. BLASTp analysis of suramin-bound protein structures across representative taxonomic groups.

## Abbreviations

AAC	ADP/ATP carrier
AGC	Aspartate/glutamate carrier
APC	ATP-Mg/Pi carrier
ATP	Adenosine triphosphate
BMEC	Brain microvascular endothelial cell
CBX	Chromobox protein
CRAT	Carnitine O-acetyltransferase
CRL	Cullin–RING ligase
DKD	Diabetic kidney disease
EGF	Epidermal growth factor
FGF	Fibroblast growth factor
GPCR	G protein-coupled receptor
HPSE	Heparanase
IMS	Intermembrane space
ITC	Isothermal titration calorimetry
LCMV	Lymphocytic choriomeningitis virus
LRP1	Low-density lipoprotein receptor-related protein 1
MIM	Mitochondrial inner membrane
MOM	Mitochondrial outer membrane
NAD^+^	Nicotinamide adenine dinucleotide (oxidized form)
NF-κB	Nuclear factor kappa B
NF-Y	Nuclear transcription factor Y
NLRP3	NOD-like receptor family pyrin domain-containing 3
ORC	Ornithine carrier
OXPHOS	Oxidative phosphorylation
PDGF	Platelet-derived growth factor
PLA_2_	Phospholipase A_2_
P2X	P2X purinergic receptor
P2Y	P2Y purinergic receptor
RyR2	Ryanodine receptor 2
SDH	Succinate dehydrogenase
SIRT5	Sirtuin 5
TAM	Tumor-associated macrophage
TCA	Tricarboxylic acid cycle
TGFβ	Transforming growth factor beta
TIMP3	Tissue inhibitor of metalloproteinases 3
TNFα	Tumor necrosis factor alpha
TRIM21	Tripartite motif-containing protein 21
VSG	Variant surface glycoprotein
