# Supplementary material for: Suramin Interactions Across Biological Systems: From Molecular Targets to Therapeutic Implications
Source: Biomolecules. 2026 Apr 1;16(4):527. doi: 10.3390/biom16040527 (PMC13113060; doi:10.3390/biom16040527)
Supplement: Supplementary file 1 [file biomolecules-16-00527-s001.zip › biomolecules-4174915-supplementary.pdf]

biomolecules

MDPI

Table S1. BLASTp analysis of suramin-bound protein structures across representative taxonomic groups. For each structure, the closest homologs identified by BLASTp are reported for bacteria, viruses, *Arabidopsis thaliana*, *Saccharomyces cerevisiae*, *Homo sapiens*, and *Trypanosoma brucei*. Cell colors indicate functionally/structurally related proteins.

**PDB ID**	**Protein Name**	**Protein Classification**	**Organism**	**Protein Lenght**	**Co-Factor**	**Ions**	**Results from BlastP analysis**				
							**Protein**	**Organism**	**Length**	**Accession number**	**% identity**
**6Z7B**	Variant Surface Glycoprotein VSGsu	Membrane Protein	*Trypanosoma brucei rhodesiense*	491	No		Variant Surface Glycoprotein Sur	*Trypanosoma brucei rhodesiense*	491	ATI14856.1	100
							Variant Surface Glycoprotein 522	*Trypanosoma brucei*	493	AGH61081.1	72

**4J4V**	Pentameric SFTSVN	Viral Protein	Phlebovirus JS2010-018	248	No		Mag: nucleocapside protein partial	*Bandavirus dabiense*	256	WFD55785.1	100
							Nucleocapside protein	*Guertu virus*	245	YP_009666940.1	89
							Nucleocapside protein	*Huaiyangshan virus*	200	AFB82711.1	99
							Nucleocapside protein	*sever fever with thrombocytopenia syndrome virus*	174	WGO58271.1	100
							Nucleocapside protein	*Heartland virus*	245	YP_010839714.1	62
**7YET**	CHAIN A: EBOV-L-VP35 (RNA-directed RNA polymerase L)	Viral Protein	Ebola Virus	2,212	No		RNA-dependent RNA polymerase	*Zaire ebolavirus*	2212	NP_066251.1	99
							RNA-dependent RNA polymerase	*Bundibugyo virus*	2210	YP_003815440.1	79

							Polymerase	*Tai Forest ebolavirus*	2210	AWK96630.1	79
							Polymerase	*Bombali virus*	2210	URC21830.1	75
							RNA-dependent RNA polymerase	*Reston ebolavirus*	2212	NP_690587.1	74
							Polymerase	*Zaire ebolavirus*	2212	QNL27324.1	89
	Chain B,C,D,E Polimerase cofactor VP35						polymerase complex protein	*Zaire ebolavirus*	340	NP_066244.1	99
							VP35	*Zaire ebolavirus*	307	AND81213.1	100
							polymerase complex protein	*Tai Forest ebolavirus*	341	YP_003815424.1	80
							polymerase complex protein	*Bundibugyo virus*	341	YP_003815433.1	78
							polymerase complex protein	*Bombali virus*	341	YP_009513275.1	77

**3UR0**	murine norovirus RNA-dependent RNA polymerase	Transferase/Transferase inhibitor	Murine norovirus 1	515	No	So4	Chain A, RNA-dependent RNA polymerase	*Murine norovirus 1*	525	3UPF_A	100
							polyprotein	*Murine norovirus GV/CR10/2005/USA*	1688	ABU55612.1	98
							RNA-dependent RNA polymerase	*Norovirus Gl*	510	YP_009701444.1	60
**3GAN**	Crystal structure of gene product from Arabidopsis thaliana At3g22680	Structural GENOMICS (UNKNOWN FUNCTION)	*Arabidopsis thaliana*	157	No	Cl	RNA-directed DNA methyl@tion 1	*Arabidopsis thaliana*	163	NP_188907.2	100
							AT3G22680	*Arabidopsis thaliana*	121	AAP21198.1	100
**6CE2**	Myotoxin	Toxin	*Bothrops moojeni*	121	No		Phospholipase A2 family protein	*Stappa Indica*	161	WP_20896812.1	50

							Phospholipase A2 membrane associated precursor	*Homo Sapiens*	144	NP_000291.1	50
							group IID secretory phospholipase A2 isoform 1 precursor	*Homo Sapiens*	145	NP_036532.1	47
							group IIE secretory phospholipase A2 precursor	*Homo Sapiens*	142	NP_055404.1	42
							Phospholipase A2 membrane associated	*Pongo Abelii*	117	XP_002811441.2	51
							Phospholipase A2 family protein	*Klensiella pneumonie*	156	MCP6508225.1	45
**1Y4L**	*Bothrops asper* myotoxin II complexed with the anti-	Hydrolase	*Bothrops asper*	121	No		Phospholipase A2 family protein	*Stappa Indica*	161	WP_20896812.1	54

	trypanosomal drug										
							group IID secretory phospholipase A2 isoform 1 precursor	*Homo Sapiens*	145	NP_036532.1	50
							group IIE secretory phospholipase A2 precursor	*Homo Sapiens*	142	NP_055404.1	45
							Phospholipase A2 membrane associated precursor	*Homo Sapiens*	144	NP_000291.1	53
							Phospholipase A2 membrane associated	*Pongo Abelii*	117	XP_002811441.2	53
							Phospholipase A2 family protein	*Klensiella pneumonie*	156	MCP6508225.1	51

**4YV5**	Myotoxin II	Toxin	*Bothrops moojeni*	122	No	SO4	Phospholipase A2 family protein	*Stappa Indica*	161	WP_20896812.1	50
							Phospholipase A2 membrane associated precursor	*Homo Sapiens*	144	NP_000291.1	52
							group IID secretory phospholipase A2 isoform 1 precursor	*Homo Sapiens*	145	NP_036532.1	50
							group IIE secretory phospholipase A2 precursor	*Homo Sapiens*	142	NP_055404.1	44
							Phospholipase A2 membrane associated	*Pongo Abelii*	117	XP_002811441.2	53
							Phospholipase A2 family protein	*Klensiella pneumonie*	156	MCP6508225.1	50

**3BJW**	Ecarpholin S	Hydrolase	*Echis carinatus*	122	No		Phospholipase A2 family protein	*Stappa Indica*	161	WP_20896812.1	68
							Phospholipase A2 membrane associated precursor	*Homo Sapiens*	144	NP_000291.1	47
							group IID secretory phospholipase A2 isoform 1 precursor	*Homo Sapiens*	145	NP_036532.1	46
							group IIE secretory phospholipase A2 precursor	*Homo Sapiens*	142	NP_055404.1	45
							Phospholipase A2 membrane associated	*Pongo Abelii*	117	XP_002811441.2	48
							Phospholipase A2 family protein	*Klensiella pneumonie*	156	MCP6508225.1	47

**4X3U**	Chromobox homolog 7 (CBX7) chromodomain	Transcription	*Mus musculus*	64	No		Chromobox protein homolog 7	*Rattus norvegicus*	158	**NP_954548.1**	95
							chromobox protein homolog 7 isoform X2	*Phyllostomus discolor*	250	XP_028388249.1	97
							Chromobox protein homolog 7	*Macaca mulatta*	251	NP_001247702.1.1	97
							Chromobox protein homolog 6 isoform 1	*Homo Sapiens*	414	NP_055107.3	71
							Chromobox protein homolog 2 isoform 2	*Homo Sapiens*	211	NP_116036.1	66
							Chain A e3 sumo protein ligase CBX4	*Homo Sapiens*	60	2K1B_A	68

							E3 SUMO-protein ligase CBX4	*Homo Sapiens*	560	NP_003646.2	68
							TPA: chromobox like protein 4	*Bos Taurus*	287	DAA18114.1	68
							CBX8 isoform 3 partial	*Pongo Abelii*	173	2B28_A	75
**3PP7**	Leishmania mexicana pyruvate kinase	Transferase/Transferase inhibitor (of glycolysis)	*Leishmania mexicana*	498	No	K	Pyruvate kinase PKM isoform a	*Homo sapiens*	531	NP_002645.3	49
							pyruvate kinase PKLR isoform X1	*Nomascus leucogenys*	604	XP_030680175.1	48
							pyruvate kinase PYK2	*Saccharomyces cerevisiae S288C*	506	NP_014992.3	49
							Pyruvate kinase PKM isoform 4	*Mus musculus*	552	NP_001365801.1	49
							pyruvate kinase PKLR isoform 1	*Homo sapiens*	574	NP_000289.1	48

							Pyruvate kinase	*Allibacillus*	588	WP_212369482.1	48
							Pyruvate kinase	*Peptococcales bacterium*	583	MGI6225023.1	
**9GTE**	TRIM21 PRY-SPRY domain	Ligase	*Mus musculus*	188	No		KDA RO protein	*Homo sapiens*	181	2IWG_B	76
							E3 ubiquitin-protein ligase TRIM21	*Homo sapiens*	475	NP_003132.2	75
							E3 ubiquitin-protein ligase TRIM68 isoform 2	*Homo Sapiens*	262	NP_001291425.1	48
							erythroid membrane-associated protein isoform X4	*Homo sapiens*	396	XP_047299551.1	50
							Pyrin isoform 1	*Homo Sapiens*	781	NP_000234.1	47
							pyrin/marenostrin, partial	*Homo sapiens*	183	AAK97224.1	47
							butyrophilin subfamily 2	*Homo Sapiens*	313	NP_001184168.1	46

							member A2 isoform d precursor [				
**2NYR**	Human Sirtuin homolog 5 (NAD-dependent deacetylase sirtuin-5)	Hydrolase	*Homo Sapiens*	271	No	Zn2+	NAD-dependent protein deacylase sirtuin-5, mitochondrial isoform 1	*Pongo Abelii*	310	NP_001126552.1	99
							NAD-dependent protein deacylase sirtuin-5, mitochondrial isoform 6	*Homo Sapiens*	271	NP_001363740.1	100
							hypothetical protein H8959_011201	*Pygathrix nigripes*	235	KAL4698544.1	97
							MAG: NAD-dependent deacylase	*deltaproteobacteria bacterium HGW-*	269	PKN55970.1	50

								Deltaproteobacteria-14			
							Sir2 family NAD-dependent protein deacetylase	*Ignavibacteriaceae bacterium*	247	HEY6438107.1	62
							NAD-dependent deacylase	*Deltaprotebacteria bacterium*	266	MCB9734494.1	50
							NAD-dependent deacylase	*Planctomycetota bacterium*	272	MCB9734494.1	49
**7AH8**	NF-Y (Isoform 6 of Nuclear transcription factor Y subunit gamma subunit	Transcription	*Homo Sapiens*	80	No		nuclear transcription factor Y subunit beta isoform f	*Homo sapiens*	169	NP_001401457.1	100
							nuclear transcription factor Y subunit beta	*Equus caballus*	207	NP_001075369.2	100

							Chain B, Nuclear transcription factor Y subunit B-3	*Arabidopsis Thaliana*	103	7CVO_B	74
							NFYB/HAP3 family transcription factor subunit	*Bacterium*	164	MDA9097376.1	75
	Nuclear transcription factor Y subunit beta	Transcription	*Homo Sapiens*	89	No		NF-YC	*Homo Sapiens*	97	1N1J_B	100
							nuclear transcription factor Y subunit gamma isoform 5	*Homo sapiens*	301	NP_001136062.1	100
							nuclear transcription factor Y subunit gamma	*Xenopus tropicalis*	334	NP_989205.1	99

							nuclear factor Y, subunit C4	*Arabidopsis Thaliana*	250	NP_001032130.1	75
**3BF6**	Thrombin (light chain)	Hydrolase	*Homo Sapiens*	36	No		Chain A, Thrombin light chain	*Homo sapiens*	48	1JMO_L	100
							Chain A, Thrombin light chain	*Homo sapiens*	42	4RKO_A	100
							Chain X, Prothrombin	*Homo sapiens*	152	6PX5_X	100
							prothrombin precursor	*Pongo Abelii*	623	NP_001126851.1	100
							coagulation factor II (thrombin), isoform CRA_b	*Homo sapiens*	556	EAW67978.1	100
							F2 isoform 2	*Pongo Abelii*	584	PNJ68411.1	100
	Thrombin (heavy chain)	Hydrolase	Homo Sapiens	259	No		prothrombin preproprotein [Homo sapiens]	*Homo sapiens*	622	NP_000497.1	100
							Prethrombin-1	*Homo Sapiens*	424	3NXP_A	100

							Prothrombin precursor	*Pongo Abelii*	623	NP_001126851.1	99
							Chain B, Thrombin	*Mus musculus*	259	2OCV_B	89
**2H9T**	Human alpha-thrombin – Chain A	Hydrolase	Homo Sapiens	36	No		Chain A, Thrombin light chain	*Homo sapiens*	42	4RKO_A	100
							Chain A, Thrombin light chain	*Homo sapiens*	48	1JMO_L	100
							Chain X, Prothrombin	*Homo sapiens*	152	6PX5_X	100
							coagulation factor II (thrombin), isoform CRA_b	*Homo sapiens*	556	EAW67978.1	100
							prothrombin precursor	*Pongo Abelii*	623	NP_001126851.1	100
							F2 isoform 2	*Pongo Abelii*	584	PNJ68411.1	100

	Human alpha-thrombin – Chain B	Hydrolase	Homo Sapiens	259	No		Chain A, Prethrombin-1	*Homo sapiens*	424	3NXP_A	100
							Prothrombin precursor	*Pongo Abelii*	623	NP_001126851.1	99
							Chain B, Thrombin	*Mus musculus*	259	2OCV_B	89
